# Glucocorticoid-Mediated Skeletal Muscle Atrophy: Molecular Mechanisms and Potential Therapeutic Targets

**DOI:** 10.3390/ijms26157616

**Published:** 2025-08-06

**Authors:** Uttapol Permpoon, Jiyeong Moon, Chul Young Kim, Tae-gyu Nam

**Affiliations:** Department of Pharmacy, Institute of Pharmaceutical Science and Technology, Hanyang University ERICA, Ansan 15588, Gyeonggi-do, Republic of Korea; uttapol.pem@gmail.com (U.P.); jiyeong7202@gmail.com (J.M.); chulykim@hanyang.ac.kr (C.Y.K.)

**Keywords:** glucocorticoids, muscle atrophy, atrogenes, SIRT6, LSD1, IDO-1

## Abstract

Skeletal muscle atrophy is a critical health issue affecting the quality of life of elderly individuals and patients with chronic diseases. These conditions induce dysregulation of glucocorticoid (GC) secretion. GCs play a critical role in maintaining homeostasis in the stress response and glucose metabolism. However, prolonged exposure to GC is directly linked to muscle atrophy, which is characterized by a reduction in muscle size and weight, particularly affecting fast-twitch muscle fibers. The GC-activated glucocorticoid receptor (GR) decreases protein synthesis and facilitates protein breakdown. Numerous antagonists have been developed to mitigate GC-induced muscle atrophy, including 11β-HSD1 inhibitors and myostatin and activin receptor blockers. However, the clinical trial results have fallen short of the expected efficacy. Recently, several emerging pathways and targets have been identified. For instance, GC-induced sirtuin 6 isoform (SIRT6) expression suppresses AKT/mTORC1 signaling. Lysine-specific demethylase 1 (LSD1) cooperates with the GR for the transcription of atrogenes. The kynurenine pathway and indoleamine 2,3-dioxygenase 1 (IDO-1) also play crucial roles in protein synthesis and energy production in skeletal muscle. Therefore, a deeper understanding of the complexities of GR transactivation and transrepression will provide new strategies for the discovery of novel drugs to overcome the detrimental effects of GCs on muscle tissues.

## 1. Introduction

Muscle atrophy is characterized by a decreased muscle fiber mass and diameter. It commonly occurs as a side effect of many pathological conditions and has become a critical health issue affecting the quality of life of elderly individuals and patients with chronic diseases. It is caused by reduced protein synthesis and increased protein degradation [1]. Protein production in muscle cells primarily relies on phosphatidylinositol 3-kinase (PI3K)/protein kinase B (AKT) activation and mammalian target of rapamycin (mTOR) signaling, which are triggered by growth factors such as insulin-like growth factors (IGFs), hormones, and essential amino acids [2,3]. Meanwhile, protein catabolism is driven by multiple mechanisms. Studies have identified common mechanisms underlying different pathologies, which can be clustered as atrophy-related genes or atrogenes. For example, myostatin/activin signaling negatively regulates muscle mass through SMAD2/3 activation [4]. Trauma, sepsis, inflammation, and other oxidative stress conditions increase reactive oxygen species (ROS) production, leading to activation of nuclear factor-kappa B (NF-κB) and Forkhead Box O 3 (FoxO3) pathways [5]. Malnutrition, fasting, and immobilization directly activate Forkhead Box O family members (FoxOs)’ transcriptional activity [6,7]. All of these signals initiate transcription of atrogenes: predominantly Muscle RING Finger 1 (MuRF1), Atrogin1, and autophagy/lysosome-related genes [1]. MuRF1 and Atrogin1 expression leads to ubiquitin–proteasome formation, which digests various muscle proteins, especially myosin and α-actin. In addition, these signals indirectly suppress AKT/mTOR signaling. Phosphorylated AKT counteracts catabolic signals by preventing the nuclear translocation and function of FoxOs; therefore, the inhibition of PI3K/AKT signaling not only decreases protein synthesis rates but also facilitates protein degradation.

Excessive and dysregulated glucocorticoid (GC) production is found in the aged, chronic diseases, metabolic diseases, and endocrine disorders such as Cushing syndrome [8,9]. GC, acting through the glucocorticoid receptor (GR), initiates muscle atrophy, particularly in fast-twitch muscle fibers, in the following two ways: decreasing protein anabolism and increasing protein catabolism [10]. GCs directly suppress the PI3K/AKT signal by reducing the expression and function of insulin receptor substrate-1 (IRS-1), an early mediator in PI3K activation [11]. It also increases the expression of the mTORC1 suppressor, regulated in development and DNA damage 1 (REDD1) and Kruppel-like factor 15 (KLF-15), which further suppress protein synthesis [12]. GCs facilitate the nuclear translocation of FoxOs through AKT inactivation and promote the transcription of FoxOs, atrogenes, and myostatin, as direct targets of GR [13,14]. Furthermore, GCs also decrease glucose and calcium uptake through a GR-independent pathway, impairing glycolytic metabolism and muscle function in movement [15,16].

Recent studies have suggested that several mechanisms are involved in GC-induced muscle atrophy. The sirtuin 6 isoform (SIRT6) is upregulated in GC-treated myotubes, correlating with a reduction in myofiber size and glucose uptake [17]. A study indicates that SIRT6 blocks IGF-2 transcription. SIRT6 inhibitors increase PI3K/AKT activation and prevent muscle wasting in GC-treated myotubes. Second, lysine-specific demethylase 1 (LSD1) exhibits protein–protein interactions with GR to initiate atrogene expression [18]. LSD1 inhibition is sufficient to overcome muscle atrophy induced by GC. Tryptophan metabolism via the kynurenine (KYN) pathway also plays an important role in muscle biology; tryptophan stimulates the PI3K/AKT/mTOR axis, while KYN induces oxidative stress and proteolysis [19]. Indoleamine 2,3-dioxygenase (IDO)-1 (IDO-1), a rate-limiting enzyme converting tryptophan to KYN, is significantly upregulated in response to GCs indirectly through FoxO3 upregulation, leading to tryptophan deprivation and accumulation of KYN [20]. Taken together, multiple pathways are involved in GC-mediated muscle wasting. This review discusses the underlying mechanisms of GC-mediated muscle atrophy and potential interventions based on a search of PubMed, ScienceDirect and ClinicalTrials.gov, which was conducted using search terms related to muscle atrophy and GC to identify studies between the years 2010 to 2025.

## 2. Glucocorticoids

Signals such as low glucose levels, emotional and physiological stress, and inflammation trigger the hypothalamus to release corticotropin-releasing hormone (CRH), which stimulates the anterior pituitary gland to release adrenocorticotropic hormone (ACTH), followed by the synthesis and secretion of GCs into the bloodstream. GCs promote negative feedback to regulate the hypothalamic–pituitary–adrenal (HPA) axis [21], an important survival mechanism in humans to conserve the homeostasis of nutrient metabolism, stress adaptation, and immune response [22,23,24]. GC activity varies in various tissues depending on the presence of enzymes 11β-hydroxysteroid dehydrogenases (11β-HSDs). 11β-HSD2 is highly expressed in the kidney and pancreas, where it converts active GCs to the inactivated form, while 11β-HSD1 works in the opposite way and is primarily present in the liver, adipose tissue, brain, lung, and muscle [25]. Impairment of the HPA axis causes diverse syndromes, such as intense fatigue, prolonged stress, sepsis, and autoimmune disorders [26,27].

GCs predominantly act through GR, which is expressed in most tissues [28,29]. The GR forms complexes with heat shock proteins in an inactive state. This process requires ligand-dependent activation for translocation into the nucleus. Activated GR binds to GC response elements (GREs) and initiates gene transcription; this action is called “GR transactivation.” GREs have the sequence 5′-AGAACAnnnTGTTCT-3′ and are typically located near the transcription start sites of GC-responsive genes in a cell-specific manner. For example, they regulate glucose-6-phosphatase for gluconeogenesis in hepatocytes [30], interleukin-10 (IL-10) in B lymphocytes [31], and genes involved in tissue repair and wound healing functions in fibroblasts [32]. GCs exhibit “GR transrepression” indirectly through protein–protein interaction to block the activity of transcription factors like NF-κB and activator protein 1 (AP-1) [33]. NF-κB inhibition leads to reduced pro-inflammatory cytokine production and downregulation of cell surface molecules essential for immune functions [34]. GR-mediated repression of NF-κB-dependent inflammation is a classical mechanism underlying the immunosuppressive properties of GC.

In the 1940s, a synthetic compound E, the first version of cortisone, was used to treat a female patient with severe rheumatoid arthritis (RA) at the Mayo Clinic in the US [35]. Since then, synthetic GCs have been applied for various inflammatory and autoimmune diseases, as well as other pathological conditions, such as multiple sclerosis, inflammatory bowel disease, lupus syndromes, asthma, chronic obstructive pulmonary disease, allergies, organ transplant recipients, and even some cancers [36]. Fluorinated GCs (e.g., dexamethasone) exhibit higher stability and potency than non-fluorinated GCs (e.g., prednisone) [37]. Most commonly used GCs, such as cortisone, hydrocortisone, prednisone, methylprednisolone, dexamethasone, betamethasone, and triamcinolone, provide high potency and cost-effectiveness in a broad spectrum of pathologies. However, long-term use of GCs is associated with side effects affecting multiple organs [38]. For example, GCs elevate cholesterol, triglyceride, body weight, and visceral fat deposits, which increase the risk of cardiovascular disorders [39]. Exogenous GCs cause endocrine system imbalance and increase blood sugar, causing metabolic disorders such as obesity and diabetes [40]. Myopathy is one of the most common side effects that can occur with high dosage and chronic exposure to GCs, especially fluorinated GCs [41].

## 3. Effects of Glucocorticoids on Muscle Tissues

Human muscles highly express 11β-HSD1, a key regulatory enzyme converting inactive GC (cortisone) to active GC (cortisol); therefore, GC exerts direct effects in muscle tissues [42]. 11β-HSD1 levels in skeletal muscle increase in females with age, making women more susceptible to adverse effects than men [43]. Additionally, pro-inflammatory cytokines, particularly tumor necrosis factor-α (TNF-α), elevate 11β-HSD1 through the NF-κB pathway [44]. Several 11β-HSD1 inhibitors have been evaluated in clinical trials intensively focusing on type 2 diabetes mellitus and idiopathic intracranial hypertension [45,46]. Notably, the 11β-HSD1 inhibitor AZD4017 demonstrated improvements in lean muscle mass in a phase II study involving overweight women [47]. Similarly, it prevented adverse events caused by prednisolone in men [48].

GC levels in humans naturally peak during the day and decline in the evening [8]. However, this circadian rhythm can be disturbed by psychological and physiological stressors such as aging, neuronal disorders, inflammation, cancers, and treatment with glucocorticoids. Consequently, metabolic balance is impaired [8]. Sarcopenia or cachexia (degenerative or disease-related muscle atrophy, respectively) is frequently reported in these conditions, which are probably linked with the HPA axis and GC dysregulation. For example, more than half of the patients with Cushing’s syndrome suffer from muscle atrophy and myopathy due to ACTH hypersecretion by pituitary adenoma or ectopic sources, leading to prolonged exposure to high cortisol levels [9]. Cancer and chronic inflammation activate the HPA axis and increase GC concentration in the serum, skeletal muscle, and liver [49], leading to skeletal muscle proteolysis.

In the case of synthetic GCs, acute steroid myopathy can occur rapidly upon administration of moderate doses of GC [50]. Changes in muscle size and weight can be detected within 7 days after starting GC administration. Furthermore, fiber cross-sectional area and myofibril content decrease significantly. GCs interrupt muscle protein synthesis and facilitate protein catabolism, resulting in decreased muscle mass and strength. Fast-twitch type II fibers and glycolytic muscles such as the biceps, triceps, quadriceps, and hamstrings are particularly susceptible to GC, whereas slow-twitch type I fibers, oxidative muscles, and cardiac muscles are more resistant to GC [51,52]. This may be primarily because GRs are more abundant in fast-twitch fibers than in slow-twitch fibers [53]. Another factor is a source of energy. Anaerobic glycolytic muscles rely on stored glucose in the form of glycogen, thereby highly sensitive to GC-induced downregulation of GLUT4 and inhibition of glycogen synthesis in skeletal muscle [54]. In contrast, slow-twitch fibers have a high density of blood vessel supply and primarily use aerobic metabolism which provides much ATP from glucose consumed from blood [55]. Fast-twitch fibers also show more pronounced changes in calcium homeostasis [56]. Furthermore, slow-twitch fibers naturally achieve protection from peroxisome proliferator-activated receptor-γ coactivator-1α (PGC-1α) that can enhance glucose transporter, oxidative phosphorylation, fatty acid oxidation, and mitochondrial functions [57]. Endurance exercise, β-adrenergic stimulation, vitamin A supplementation, and dietary oleic acid intake have been reported to increase PGC-1α expression in fast-twitch muscle by supporting muscle adaptation [58,59,60].

Thus, the development of endogenous and prolonged exogenous GC-associated myopathy is a problem in many patients, including in an aging society. Although this issue has been addressed for several years, there are still only a few effective ways to overcome it.

## 4. Molecular Mechanisms of Glucocorticoid-Induced Muscle Atrophy

**Figure 1:**
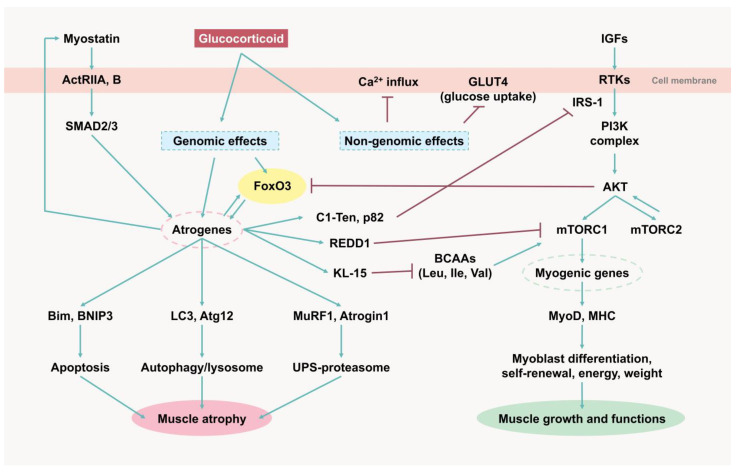
Molecular mechanisms of glucocorticoid-induced muscle atrophy. ActRIIA, B, activin type II receptor A and B; GLUT4, glucose transporter 4; IGFs, insulin-like growth factors; RTKs, receptor tyrosine kinases; IRS-1, insulin receptor substrate-1; PI3K, phosphatidylinositol 3-kinase; AKT, protein kinase B (PKB); mTORC, mammalian target of rapamycin complex; MyoD, myoblast determination protein 1; MHC, myosin heavy chain; FoxO3, Forkhead Box O 3; REDD1, regulated in development and DNA damage 1; KLF-15, Kruppel-like factor 15; BCAAs, branched-chain amino acids; MuRF1, Muscle RING Finger 1; Atrogin1, Muscle Atrophy F-box (MAFbx).

GCs alter the metabolic profile, enhance muscle protein catabolism, and suppress protein synthesis through multiple mechanisms, both genomic and non-genomic (Figure 1). Several inhibitors and blockers have been developed to counteract the adverse effects of GCs on muscle tissue based on the following molecular mechanisms (Table 1).

### 4.1. GLUT4

Skeletal muscle is a major source of glucose storage in a stable complex branched form called glycogen [71]. To maintain and increase blood glucose, GCs facilitate glycogenolysis and suppress glucose uptake and glycogen synthesis in muscle cells [72]. Acute and chronic GC exposure tends to induce insulin resistance and impair glucose uptake, among other effects [73]. Dexamethasone inhibits the translocation of glucose transporter 4 (GLUT4) from the cytoplasm to the cell membrane, without altering GLUT4 levels in rat muscle cells [16]. Electrical pulse-stimulated C2C12 myotubes exhibit decreased contraction after 20 min of incubation with dexamethasone [74]. The phosphorylation of adenosine monophosphate-activated protein kinase (AMPK) and activation of calcium/calmodulin-dependent protein kinase II (CaMKII), which are required for GLUT4 translocation, are significantly reduced by GR-independent GC action [74]. Similarly, basal Ca^2+^ influx and murine airway smooth muscle (ASM) tone is reduced after 10 min exposure to dexamethasone [15]. This rapid non-genomic response of muscle cells to GCs is not blocked by the genomic-mediated action of GR antagonists (Mifepristone (RU486) or actidione) [15,74].

### 4.2. PI3K/AKT/mTOR

PI3K, AKT, and mTOR are key molecules in cellular responses and protein synthesis, forming a complex signaling cascade [75,76,77]. Briefly, insulin or IGF-1 binds to cell-surface receptor tyrosine kinases (RTKs) and initiates tyrosine-phosphorylation of IRS-1. Phosphorylated IRS-1 induces the recruitment of the PI3K complex to the cell membrane and activates p110 by relieving the inhibitory effects of p85. Subsequently, p110 converts phosphatidylinositol biphosphate (PIP2) to phosphatidylinositol triphosphate (PIP3). PIP3 recruits 3-phosphoinositide-dependent protein kinase-1 (PDK-1) and AKT to the membrane. Furthermore, AKT is phosphorylated by PDK-1, which continuously activates mTOR function through the inactivation of tuberous sclerosis complex 1/2 (TSC1/2) and accumulation of the small G-protein Rheb-GTP. mTOR complex 1 (mTORC1) then initiates ribosomal protein S6 activity in protein synthesis by phosphorylation of S6K1 and 4EBP1, and releases the eukaryotic translation initiation factor eIF-4E. Phosphorylated AKT directly inhibits glycogen synthase kinase 3 beta (GSK3β), an eIF-4E suppressor. mTORC2 amplifies the signal by phosphorylating AKT via a positive feedback loop. In addition to the PI3K/AKT axis, mTOR senses mechanical stimulation and amino acids through an IGF-1-independent cascade. These steps result in the transcription of essential proteins that construct muscle fibers.

GCs induce muscle atrophy by inhibiting PI3K/AKT and mTORC1 signaling via multiple mechanisms. GR knockdown attenuated GC-mediated IRS-1 reduction, indicating that IRS-1 is a direct target of GRs [78]. Dexamethasone-treated C2C12 cells and chronic corticosterone-treated mice exhibited elevated inactive pSer307-IRS-1 levels, which were neutralized by GR blocker and 11β-HSD1 inhibitor, respectively [11]. The expression of the IRS-1 tyrosine phosphatase C1-Ten was also upregulated by GC [79]. Dexamethasone-induced p85α upregulation was strongly associated with insulin resistance and myotube size reduction [80,81]. Dexamethasone and myostatin induced microRNA1 (miR1) overexpression in C2C12 cells [82]. miR1 downregulates heat shock protein 70 (HSP70) which helps stabilize phosphorylated AKT, suppressing downstream AKT signaling.

GCs mediate mTORC1 inhibition by upregulating mTORC1 suppressors, including REDD1 and KLF-15 as direct targets of activated GRs [12]. Consistently, REDD1 induction is also found in metabolic-related pathological conditions, such as oxidative stress, diabetes, cancer, and hypoxia [83,84]. This phenomenon is called mTORC1 suppression via TSC2 activation [85]. The crystal structure of REDD1 revealed its binding sites for phospho-binding 14-3-3 proteins on TSC2 [86]. High levels of REDD1 are correlated with GC-induced muscle atrophy. Moreover, REDD1 knockout preserved the size and weight of leg muscles in mice subjected to prolonged dexamethasone treatment, while maintaining the anti-inflammatory properties of GC [87,88]. These findings suggest that REDD1 is a key factor in GC-induced muscle atrophy. Inhibition of REDD1 may prevent adverse events but retain the benefits of GC. Currently, specific REDD1 inhibitors are not available. However, a research group suggested that some pharmacological PI3K/AKT inhibitors reduced REDD1 expression in normal physiology and GR-mediated REDD1 upregulation even while potentially suppressing the mTOR pro-proliferation effect [89,90,91]. They demonstrated that PI3K inhibitors, rapamycin and LY294002, prevented GC-mediated skin atrophy and synergized well with GCs in blood cancer treatment by blocking GR trans-activation and NF-κB activity but enhancing GR trans-repression [89,90,91]. Although its mechanism is unclear, the researchers hypothesized that this response may underline the feedback loop of AKT/mTOR signaling and GR post-translational modification. In contrast, another study showed that rapamycin significantly upregulated the expression of REDD1, KLF15, Atrogin1, MuRF1, FoxO1, and FoxO3a in rat L6 myotubes [12]. Auto-stimulation of mTOR attenuated the transcription of these genes. These results indicate that mTOR signaling negatively regulates GR transactivation genes; therefore, the inhibition of mTOR is thought to derepress atrogenes. Another hypothesis is the unidentified off-target effects of PI3K/AKT inhibitors. For instance, LY294002 binds to BET proteins (Bromodomain and Extra-Terminal motif proteins) that are involved in histone acetylation [92]. Rapamycin may alter the translation of nutrient-sensing 5′ terminal oligopyrimidine motif containing mRNA which is regulated by both mTOR-dependent and -independent mechanisms [93,94]. Nevertheless, the connection of those mechanisms with REDD1 remains to be elucidated. Therefore, selective REDD1 inhibitors are required to prevent unpredictable responses.

Unlike REDD1, KLF-15 indirectly regulates mTOR by modulating the catabolism of branched-chain amino acids (BCAAs: leucine, isoleucine, and valine). BCAAs are not only essential amino acids for building muscle tissue but also key factors in cellular signaling transduction [95]. Leucine and isoleucine enhance glucose uptake in C2C12 myotubes and rat muscle by elevating expression and membrane translocation of GLUT1 and GLUT4 [96,97,98]. Leucine stimulates skeletal muscle fiber regeneration after exercise [99]. Several studies have demonstrated that leucine initiates protein synthesis and increases muscle mass via direct mTOR activation [100,101,102,103]. Leucine supplementation also improves pork texture [104] and milk products [105]. KLF-15 is a member of the zinc-finger transcription factor KLF family, which is involved in various cellular functions [106,107]. In skeleton muscle, GR drives the induction of KLF-15, and then GR and KLF-15 coordinately initiate the transcription of branched-chain amino acid aminotransferase 2 (BCAT2) [12]. BCAAs are converted into branched-chain α-ketoacids by BCAT2 and further catabolized by other enzymes to products entering the TCA cycle or secreted extracellularly [95]. KLF-15 induction facilitates BCAAs catabolism leading to alanine secretion and leucine deficiency in muscle cells [12]. Moreover, KLF-15 upregulates Atrogin-1, MuRF1, FoxO1, and FoxO3a, which further enhance protein degradation. A BCAAs cocktail restores mTOR signaling and muscle strength in the presence of dexamethasone. In contrast, transient overexpression of KLF-15 inhibits cardiac hypertrophy by repressing GATA4, MEF2, and myocardin [108]. As mentioned above, KLF-15 plays a crucial role in GC-related muscle atrophy. Unfortunately, the development of KL-15 inhibitors is challenging because of the lack of information regarding their interaction domains [106].

### 4.3. Myostatin

Myostatin, also known as growth differentiation factor-8 (GDF-8), is a member of the transforming growth factor-β (TGF-β) superfamily, which is produced and secreted into the bloodstream by mature muscle fiber and acts as a negative regulator of muscle growth [109]. Myostatin controls the balance between protein synthesis and degradation by regulating AKT/mTOR signaling and proteasome activity [110,111]. Myostatin helps to protect cardio myofiber overgrowth following stress and prevent glycogen accumulation, which can cause cardiovascular diseases [112]. Myostatin-deficient mice develop hyperplasia and hypertrophy of skeletal muscle [113].

Myostatin interacts with surface activin type II receptors A and B (ActRIIA, B), which recruit activin receptor-like kinase receptors (ALKs) to phosphorylate transcription factors SMAD2 and SMAD3 [114]. Sequentially, activated SMAD2 and SMAD3 form a complex with SMAD4 and translocate inside the nucleus to regulate gene expression [115,116]. Myostatin/ActRII signaling reduces the phosphorylation of key mediators in the AKT/mTOR pathway and protein synthesis [117]. Myostatin decreases the expression of miR-486, which targets the AKT repressor, phosphatase and tensin homolog (PTEN). Therefore, miR-486 supports AKT activation in muscle cells [118,119]. Alternatively, the activation of mTORC1 prevents self-amplification of myostatin signaling [120]. Furthermore, SMAD2/3 induces expression of MuRF1 and Atrogin1, which lead to protein degradation via proteasome formation [121]. Myostatin also suppresses myoblast proliferation increasing p21, a cyclin-dependent kinase inhibitor [122].

Myostatin overexpression strongly corresponds to various conditions including stress, obesity, chronic metabolic diseases, and immobility. Myostatin production is upregulated by several factors, including oxidative stress, inflammation, uremic toxins, metabolic acidosis, low physical activity, and hormones, including angiotensin II and GCs. Dexamethasone increases the levels of intramuscular myostatin mRNA and protein correlating with muscle loss in a dose-dependent manner in a rat model [123]. GREs, present at the promotor of the myostatin gene, can be blocked by RU486 [13]. Myostatin-null mice are resistant to muscle atrophy induced by prolonged dexamethasone treatment [124]. Dexamethasone-treated C2C12 cells show reduced total protein synthesis, and high levels of myostatin, accompanied by decreased p70S6K, and increased ubiquitination-associated proteins [125]. Myostatin plays a crucial role in GC-induced muscle atrophy and may be resolved by myostatin inhibition. Various myostatin blockers have been developed using diverse platforms, including intact or partial antibodies, soluble decoy ActRII, propeptides, natural compounds, and the endogenous myostatin antagonist follistatin. These blockers have demonstrated potent effects in preventing muscle atrophy associated with chronic disorders, hereditary diseases and prolonged GC treatment in pre-clinical phases [126,127]. Unfortunately, many of them have been terminated from clinical trials due to a lack of therapeutic potential, which raises concern about the translation of current animal models for muscle-related diseases [128]. Some myostatin inhibitors could also exhibit non-specific targets to other TGF-β family members, such as bone morphogenetic protein 9 and 10 (BMP9/10), the inhibition of which can lead to muscle and bone deformities [129]. This may be the primary reason why these inhibitors have not demonstrated sufficient functional improvement in clinical setups. Furthermore, a study on Duchenne muscular dystrophy (DMD) demonstrated that GCs counteracted myostatin inhibitors and exhibited an atrophic phenotype [130]. Thus, myostatin inhibitors may not be suitable for the prevention of GC-induced muscle atrophy.

Another option is an anti-ActRII antibody, such as bimagrumab, to specifically block the binding of both myostatin and activin A [65]. It improves muscle weakness and atrophy caused by GCs in pre-clinical settings and demonstrates some benefits in various indications, including chronic obstructive pulmonary disease, obesity, and type 2 diabetes mellitus patients [66,131]. In contrast, it significantly reduces fat accumulation and body weight and poorly improves muscle functional capacity and mass [131]. However, it may also share some side effects with myostatin inhibitors. Additionally, sotatercept, a decoy of ActRIIa which binds to activin A and other ligands in the TGF-β superfamily, has been recently approved to treat patients with pulmonary arterial hypertension, even though its effect in skeletal muscle remains unclear [132]. Currently, assessment of the impact of ActRII blockers on muscle wasting and bone fractures is ongoing. Hopefully, this will attenuate muscle loss in patients receiving GCs.

### 4.4. Forkhead Box O

Forkhead Box O (FoxO) is a family of protein transcription factors composed of FoxO1, FoxO3 (the same as FoxO3a), FoxO4, and FoxO6 [133,134]. FoxOs play important roles in metabolism, stress, aging, longevity, mitochondrial function, cell cycle, and apoptosis. In terms of muscle homeostasis, FoxO1 and FoxO3 introduce myofiber protein degradation through the ubiquitin–proteasome system (UPS) and autophagy/lysosome complex [135,136]. GC-treated myotubes exhibit elevated FoxO1/3 expression, while the GR antagonist RU-486 partially neutralized this effect [137,138]. GRE enrichment is present at the promoter of FoxO1/3, indicating that the GR directly promotes FoxO1 and FoxO3a mRNA transcription. Furthermore, the GR may facilitate FoxOs transcription via calcium-dependent systems [139]. The stability and activity of FoxOs are regulated by posttranslational modifications. Activated AKT blocks FoxO functions through phosphorylation at three specific sites (Thr32, Ser253, and Ser315 for FoxO3) [140,141]. This phosphorylation disturbs FoxO interaction with the FoxO response element (FRE), leading to FoxO exclusion from the nucleus and cytoplasmic retention through 14-3-3 chaperone protein binding [142]. GC-mediated AKT suppression promotes the nuclear translocation of FoxO1/3 and the transcription of target genes [143]. Stimulation of PI3K/AKT signaling is sufficient to prevent GC-induced FoxO1/3 upregulation and muscle atrophy [141].

FoxOs promote UPS-mediated protein degradation by upregulating E3 ubiquitin ligases and several related proteins that form proteasome complexes. FoxO3 is the primary transcription factor driving the expression of most atrogenes in early response to GC treatment [14]. FoxO3 directly binds to the promotor of muscle atrophy F-box (MAFbx) known as Atrogin1 [144,145]. FoxO3 expression is correlated with dose-dependent MuRF1 upregulation; however, DNA binding domain-mutated FoxO3 only slightly affects the transcription of MuRF1, suggesting that FoxO3 may increase MuRF1 levels through DNA-binding-independent mechanisms [146]. SMAD3 binds to the proximal region of the MuRF1 promotor and it is required for FoxO3-induced MuRF1 expression [144]. This suggests that SMAD3 is an important partner of FoxO3. FoxO1 alone and in combination with the GR can upregulate MuRF1 but not Atrogin1 [147]. Moreover, FoxOs induce the transcription of ubiquitination-related proteins and proteasome subunits, including ubiquitin, MUSA1, SMART, Fbxo31, USP14, Ube4b, Itch, HDAC6, PSMD11, PSME4, PSMA1, and PSMC4 [148,149]. MUSA1, SMART, Fbxo31, and Itch are closely associated with skeletal muscle atrophy [148].

FoxOs regulate autophagy and the lysosomal complex. FoxO3 controls the transcription of autophagy-related genes, such as Bnip3, Gabarapl1, LC3, P62, Cathepsin L, PINK, ULK1, and Atg12, in skeletal muscle cells [149,150]. Autophagy is required for protein turnover and recycling to maintain the quality of muscle fibers; however, dysregulation of autophagosomes causes muscle mass loss in GC-treated myotubes and other pathological conditions of muscle atrophy [151,152].

FoxOs also regulate the metabolic switch in muscle cells [136,153]. FoxO1 increases pyruvate dehydrogenase kinase 4 (PDK4), which reduces the activity of pyruvate dehydrogenase (PDH) and inhibits the pyruvate dehydrogenase complex (PDC), resulting in a reduction in the use of carbohydrates as energy substrate. FoxOs upregulates lipoprotein lipase (LPL), fatty acid translocase/cluster of differentiation 36 (FAT/CD36), and adiponectin receptor (AdipR) transcription, enhancing fatty acid oxidation. FoxO1 inhibits peroxisome proliferator activator receptor γ coactivator-1 α (PGC-1α) and CaMKII, which negatively affect type I fiber expression. Therefore, GC-mediated FoxO activation reduces the consumption of glucose, while shifting toward the use of fatty acid as energy source in the muscle tissue.

Furthermore, GCs induce cell death via FoxO3-dependent apoptosis through the transcription of Bim (pro-apoptotic BH3-only proteins), BNIP3, and FasL genes, resulting in increased caspase-3 activity [154].

The FoxO family members enhance their own expression. FoxO1 can induce its expression, whereas FoxO3 enhances FoxO1 and FoxO4, which further amplify target gene transcription to facilitate protein hydrolysis [134]. Thus, FoxOs, particularly FoxO1 and FoxO3, play a critical role in the cellular response to GC in the development of muscle atrophy. The FoxO1 selective inhibitor AS1842856 prevents the induction of MuRF1, increases protein synthesis via p70S6k phosphorylation, and supports fast-twitch type muscle fiber [67]. A potent FoxO3 inhibitor, carbenoxolone, has been tested to solve drug resistance in neuroblastoma [69]. Although there are currently few options for FoxO3 inhibition and no direct experiments in a muscle atrophy model, FoxO3 inhibitors hold promise to serve as potent drugs for preventing the proteolytic effect of GCs in muscle.

### 4.5. Atrogenes and UPS-Mediated Protein Degradation

The UPS plays a critical role in protein degradation, causing myofibers to lose weight and strength [155]. Ubiquitination is a post-translational protein modification involving a chain reaction of enzymes and multiple subunits: E1 (ubiquitin-activating enzyme), E2 (ubiquitin-conjugating enzyme), and E3 (ubiquitin ligase). Polyubiquitination initiates the formation of the proteasome complex, which is a barrel-shaped structure with proteolytic activity.

MuRF1 and Atrogin1 are muscle-specific E3 ubiquitin ligases involved in muscle atrophy [156]. Even though their functions are the same, they have different structures and distinct targets. MuRF1 contains a RING finger domain, but Atrogin1 is an F-box type ligase [155]. MuRF1 primarily targets contractile and structural proteins: myosin heavy chain (MHC), myosin light chain (MLC), α-actin, and telethonin [157,158]. In contrast, Atrogin1 interacts with regulatory proteins involved in muscle differentiation, growth, and metabolism, such as MyoD and eukaryotic translation initiation factor 3 subunit F (eIF3-f) [159,160,161]. In general, the expression of MuRF1 and Atrogin1 are regulated by multiple signals based on nutrient availability and hormones [162]. GCs increase MuRF1 and Atrogin1 expression in various ways. GC-induced FoxO activation leads to the induction of MuRF1 and Atrogin1 [14]. FoxO1 exclusively transcribes MuRF1, while FoxO3 is sufficient to activate both MuRF1 and Atrogin1 transcription [147]. Silencing CCAAT/enhancer binding proteins (C/EBPβ) attenuated dexamethasone-induced MuRF1 and Atrogin1 upregulation [163]. MicroRNA-23a (miR-23a), a negative epigenetic regulator of MuRF1 and Atrogin1, is downregulated in response to GC treatment through inhibition of the calcineurin-NFAT signal [164,165]. GC-induced Notch signaling activation is also involved in upregulating atrogenes, exclusively in muscle cells [50,166]. GC trans-repression inhibits the activity of NF-κB representing anti-proliferation and anti-inflammation in cancer cells and immune cells, respectively [167,168]. In contrast, GCs promote NF-κB-inducing kinase (NIK) activating NF-κB to transcript MuRF1 and Atrogin1 along with myostatin and Gadd45 [169]. Additionally, GC-induced myostatin secretion also supports atrogene expression [125].

These atrogenes are expressed in all muscle types. However, some studies have suggested that MuRF1 and Atrogin1 have different expression patterns in each myofiber type. For instance, the upregulation of MuRF1 and Atrogin1 is faster and higher in fast-twitch muscle than in slow-twitch muscle in response to immobilization-induced atrophy [170]. Even though it has not been clarified in the case of GC-induced atrophy, muscle-specific regulation may be represented in most cases of muscle atrophy.

As mentioned above, atrogenes, particularly MuRF1 and Atrogin1, are critical components of decaying myofibers. Although it is difficult to directly block their activity, the inactivation of these genes through the manipulation of upstream mechanisms is beneficial for preventing atrophy induced by GC. Interestingly, activation of Vitamin D receptor (VDR) signaling with VDR ligands could repress MuRF1 and maintain muscle mass in the presence of GC, although the exact molecular mechanism is not clear [70]. Vitamin D promotes skeletal muscle regeneration; in contrast, VDR knockdown impairs mitochondrial functions and myogenesis without affecting protein synthesis or anabolic signaling [171,172].

## 5. Emerging Targets for Drug Discovery

Recently, several novel pathways and targets have been identified for their crucial roles in GC-induced muscle atrophy, and the potential of their inhibitors has been demonstrated (Table 2).

### 5.1. SIRT6

SIRT6 is an emerging target in various diseases, including diabetes, aging-associated diseases, immune-related disorders, and malignancy [178]. In pre-clinical settings, SIRT6 inhibitors exhibit anti-inflammatory and anti-cancer effects and improve glucose tolerance. A phase Ib clinical trial of a small molecule SIRT6 inhibitor (IMU-856) demonstrated good tolerance and benefits in celiac disease, an inherited autoimmune disorder in which the immune system overreacts to gluten-containing food and destroys intestinal barrier integrity [179]. SIRT6 is defined as an NAD+-dependent lysine deacetylase, deacetylase, and mono-ADP-ribosyl modifier, which acts on histone (e.g., H3K9ac, H3K56ac) and non-histone proteins (e.g., transcription factors) [180]. It epigenetically regulates several genes and impacts various cellular processes, including glucose metabolism, protein synthesis, inflammation, and DNA repair. SIRT6 knockout mice develop aging phenotypes, severe hypoglycemia, loss of adipose tissue, and lymphopenia [181]. SIRT6 overexpression increased the lifespan of male mice via IGF signaling inhibition [182] and restored the balance of systemic metabolism [183]. SIRT6 downregulation has also been correlated with heart failure and cardiac hypertrophy in mice [184].

**Figure 2:**
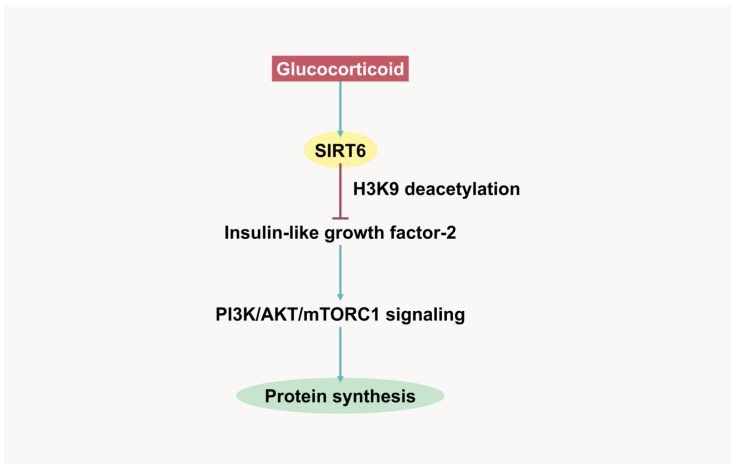
Glucocorticoid-induced SIRT6 expression in muscle protein synthesis suppression. SIRT6, Sirtuin 6; H3K9, histone 3 lysine 9; PI3K, phosphatidylinositol 3-kinase; AKT, protein kinase B (PKB); mTORC1, mammalian target of rapamycin complex 1.

A recent study demonstrated the role of SIRT6 in the regulation of muscle protein synthesis through the suppression of growth factor signaling (Figure 2). Dexamethasone-treated C2C12 cells showed significantly increased SIRT6 expression [17]. SIRT6 induction negatively correlates with myotube size and muscle mass in mice subjected to dexamethasone treatment. Furthermore, muscle-specific SIRT6 gene deletion and a pharmacological SIRT6 inhibitor protect muscles from dexamethasone-induced atrophy by enhancing IGF/PI3K/AKT signaling and reducing the expression of atrogene transcription factors FoxO1/3 [17]. This in vivo study indicated that SIRT6 deacetylates H3K9 at the IGF-2 promoter, which blocks its binding to the transcription factor c-Jun [17]. Therefore, SIRT6 depletion and inhibition allow IGF-2 transcription to activate RTKs and initiate AKT signaling. In addition, surface GLUT1 and glucose uptake are upregulated in SIRT6 inhibitor-treated rat L6 myoblasts [185]. SIRT6 activation increases FoxO3 levels and reduces GLUT1 levels in colon cancer Caco-2 cells [186]. Furthermore, SIRT6 activation introduces metabolic shifting toward mitochondrial oxidative phosphorylation [187]. In contrast, SIRT6 inhibition enhances GLUT4 levels and glycolytic metabolism in mouse muscle tissues, which directly benefits fast-twitch fibers [173]. Inactivation of SIRT6 also attenuates myofiber damage in DMD animal models [188]. Thus, SIRT6 is a promising target for preventing muscle wasting, particularly in GC-associated muscle atrophy.

### 5.2. LSD1

LSD1 is the first identified histone lysine demethylase that exhibits flavin adenine dinucleotide (FAD)-dependent amine oxidase activity [189]. It represses or activates genes depending on the specific target and protein–protein interactions. Typically, H3K4 demethylation blocks enhancer binding to the transcriptional complex, whereas H3K9 demethylation activates gene expression. Unlike most cases of histone demethylation, which frequently leads to targeted gene downregulation, LSD1, in cooperation with nuclear hormone receptors, usually initiates gene activation [190]. For example, LSD1 interacts with androgen receptors to enhance the transcription of androgen-responsive genes by modifying H3K9 in prostate cancer [191]. LSD1 also plays a pivotal role in genes that respond to retinoic acid [192], estrogen [193], and progesterone [194].

**Figure 3:**
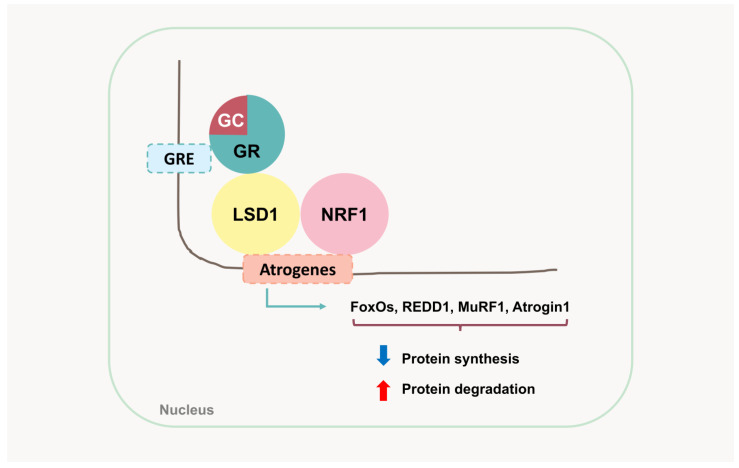
The cooperation of GR, LSD1, and NRF1 in the activation of atrogenes and enhancement of protein degradation. GRE, glucocorticoid response element; GC, glucocorticoid; GR, glucocorticoid receptor; LSD1, lysine-specific demethylase 1; NRF1, nuclear respiratory factor 1; FoxOs, Forkhead Box O members; REDD1, regulated in development and DNA damage 1; MuRF1, Muscle RING Finger 1; Atrogin1, Muscle Atrophy F-box (MAFbx).

LSD1 plays a crucial role in GC-mediated muscle proteolysis (Figure 3). In addition to being a steroid hormone receptor, the GR works closely with LSD1 to regulate multiple pathways at both normal physiological levels of GC and synthetic GC administration [18]. A study in male mice muscle suggested that LSD1 directly interacts with the GR and bridges the GR-bound enhancer with nuclear respiratory factor 1 (NRF-1)-associated promoters in muscle cells. Genomic assays revealed that GR and LSD1 co-occupied several targets, including REDD1, MuRF1, and Atrogin1. Skeletal muscle-specific LSD1 and GR double knockout mice showed reduced expression of genes involved in FoxO signaling and autophagy, but increased transcription of PI3K/AKT/mTOR-related genes. Remarkably, the deletion of LSD1 in the muscle and a selective LSD1 inhibitor, CC-90011, prevented the upregulation of atrogenes and muscle wasting during starvation and prolonged dexamethasone treatment. However, CC-90011 did not inhibit the anti-inflammatory effects of dexamethasone in immune cells. LSD1 may be required only for the GR-initiated proteolysis of myofibers. Thus, LSD1 serves as an important co-regulator of GCs.

Several LSD1 inhibitors are being investigated in clinical trials aimed at treating neurological conditions and various cancers [195,196]. Some of these inhibitors, such as CC-90011 and S2101, target the FAD cofactor-binding pocket of LSD1, which interferes with the demethylase activity of LSD1 in various histone and non-histone proteins. More identified targets and mechanisms of LSD1 will be helpful in understanding and predicting the response of muscle fibers to LSD1 inhibitors. Additionally, a greater variety of LSD1 inhibitors should be tested in muscle atrophy models. Hence, LSD1 serves as a regulator of multiple pathways in muscle biology and may be a promising target for protecting muscle fibers from the undesirable effects of GCs.

### 5.3. Kynurenine Pathway and IDO-1

**Figure 4:**
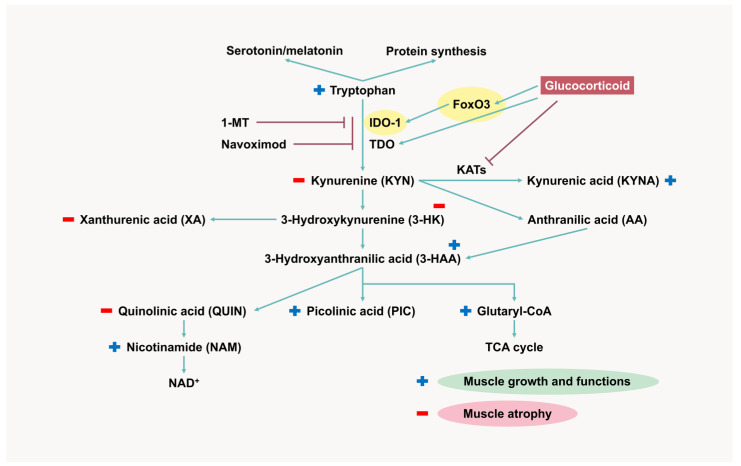
Role of the kynurenine pathway in muscle biology and effects of glucocorticoid on kynurenine metabolites. FoxO3, Forkhead Box O 3; IDO-1, indoleamine 2,3-dioxygenase 1; TDO, tryptophan 2,3-dioxygenase; KATs, KYN aminotransferases; NAD, nicotinamide adenine dinucleotide; TCA, tricarboxylic acid cycle; 1-MT, 1-methyl-tryptophan.

The KYN pathway is a tryptophan catabolism pathway composed of enzymatic and non-enzymatic reactions to produce KYN products [197]. IDO-1 catalyzes the first step that converts tryptophan to KYN, thereby being a rate-limiting enzyme of the KYN pathway [198]. The tryptophan and KYN pathways have been studied for aging, immunity, and health span implications [199,200,201]. They are also involved in muscle biology in many ways (Figure 4) [202].

The KYN substrate tryptophan is an essential amino acid [203]. It is an important amino acid and a precursor of serotonin, melatonin, and vitamin B3 (niacin) [204,205,206]. Tryptophan supplementation enhances PI3K/AKT/mTOR signaling by increasing basal levels of IGF-1, PIK3CA, TOR, 4EBP1, and S6K1 in catfish [207]. Tryptophan induces the expression of myogenic markers (MyoD, myogenin, and MHC) and follistatin, a myostatin antagonist [208]. Serum tryptophan positively correlates with skeletal muscle volume [209]. Tryptophan deficiency reduces C2C12 myoblast proliferation and differentiation. It also causes a reduction in muscle fiber diameter, weight, and strength in mice. Skeletal muscle damage in coronavirus disease 2019 (COVID-19) and cachexia in cancers are directly linked to low levels of plasma tryptophan [210,211]. Thus, tryptophan promotes muscle growth, differentiation, and function.

KYN triggers reactive oxygen species (ROS) production, leading to oxidative stress and proteolysis in muscle cells [212]. KYN administration impairs mitochondrial oxidative phosphorylation and muscle protein synthesis, leading to muscle atrophy and functional decline in mice [213]. It also elevates lipid peroxidation in muscle tissues [212]. KYN is associated with frailty and reduced physical function [213]. KYN binds to its intracellular receptor, the aryl hydrocarbon receptor (Ahr), to activate the transcription of many genes, including pro-inflammatory cytokines and IDO-1; therefore, KYN can form a positive feedback loop [214].

KYN metabolites, including kynurenic acid (KYNA), 3-hydroxykynurenine (3HK), 3-hydroxyanthranilic acid (3-HAA), anthranilic acid (AA), quinolinic acid (QUIN), picolinic acid (PIC), and xanthurenic acid (XA), have diverse effects on muscle [199]. Their synthesis is modulated by many factors. KYNA supports muscle protein synthesis and activates G protein-coupled receptor 35 (GPR35), stimulating lipid metabolism, and thermogenic and anti-inflammatory gene expression in adipose tissues [215]. KYNA is produced from KYN by enzyme KYN aminotransferases (KATs) [216]. KAT is highly expressed in oxidative type I fibers rather than glycolytic type II fibers [217]. Physical exercise rapidly induces KYNA production by activating PGC-1α and upregulating KATs in muscle tissues [216,218]. It also decreases IDO-1 and tryptophan 2,3-dioxygenase (TDO) activity and increases NAD^+^ levels via the Preiss–Handler pathway [219,220]. Therefore, exercise prevents KYN accumulation and improves muscle fitness.

Unlike KYNA, the roles of other KYN metabolites in the muscle tissues have not been well described. 3HK and XA may contribute to ROS production and mitochondrial damage, respectively, leading to inflammation and apoptosis [221,222]. QUIN, an endogenous neurotoxin, may induce muscle catabolism [223]. QUIN can be converted to nicotinamide (NAM) by quinolinate phosphoribosyl transferase entering the Preiss–Handler pathway to produce NAD^+^ [220]. Exercise increases the expression and activity of QPRT in the liver. PIC exerts anabolic effects on bone and may indirectly support muscle [224]. The synthesis of QUIN competes with PIC, as both are converted from a mediator 3-HAA by 2-amino-3 carboxymuconic-6 semialdehyde (non-enzymatic reaction) and amino-β-carboxymuconate-semialdehyde-decarboxylase, respectively. QUIN/PIC ratio increases with age and chronic diseases [199,225]. A study on the elderly revealed the relationship between inflammaging (aging-associated inflammation) and KYN metabolites [226]. An increasing ratio of KYN/tryptophan, 3-HK/3-HAA, 3-HK/XA, QUIN/3-HAA was observed, along with a decreasing NAM/QUIN ratio correlated with induction of TNF-α and a reduction in D3-creatine muscle mass.

The expression of KYN pathway-related enzymes varies in different tissues [227]. KATs and IDO-1 are commonly found in muscle [228]. The induction of IDO-1 activity corresponding to aging, cancers, diabetes, stress, and inflammation, facilitates tryptophan degradation and negatively impacts muscle fiber [202,229]. Pro-inflammatory cytokines IL-6, IL-1, and IFN-γ activate IDO-1 in the epithelium, endothelium, immune cells, and muscles [230,231,232]. GCs reduce serum tryptophan through the activation of TDO in the liver [227,233]. GC indirectly enhances IDO-1 expression by upregulating FoxO3 [20]. It introduces the de novo synthesis of amino acid-metabolizing enzymes, including IDO-1. Blocking FoxO3 expression reverses these effects. GCs, in combination with IFN-γ, synergistically enhance IDO-1 transcription in the mouse hippocampus [234]. GC may also decrease KAT expression in muscle, which leads to a reduction of KTNA synthesis [235]. Remarkably, GC induction is usually observed in pathological conditions characterized by the disruption of the KYN pathway and promotes muscle atrophy, cancers, aging, and acute/chronic inflammation [8,36,236].

IDO-1 inhibitors, such as epacadostat, indoximod, navoximod, linrodostat (BMS-986205), AT-0174, and M4112, potentially block KYN synthesis and increase tryptophan availability [237]. As IDO-1 introduces tumoral immune resistance, IDO-1 inhibitors exert anti-tumor immunity by activating the CD8+ cytotoxic T cell and inhibiting the differentiation of suppressor cells such as regulatory T cells [238,239,240]. Unfortunately, some IDO-1 inhibitors are terminated or paused in clinical trials due to a lack of improvement in progression-free and overall survival in cancer patients [241]. Some compounds derived from natural sources exhibit IDO-1 inhibition; however, their broad-spectrum activities must be considered regarding potential bystander effects and toxicity [242].

Notably, IDO-1 inhibitors are beneficial for muscle tissues. Selective IDO inhibitor 1-methyl-tryptophan (1-MT), a tryptophan analog, increased muscle size and strength by inhibiting KYN-induced ROS production and muscle lipid peroxidation in an aging model [19,212]. 1-MT also reduced KYN accumulation by facilitating KYNA synthesis [175]. A dual IDO/TDO inhibitor, navoximod, attenuated KYN-dependent sepsis-induced muscle loss correlating with reduction of atrogene transcription and activation of AKT signaling [176]. Though none of the IDO-1 inhibitors have been tested in a GC-treated model directly, increasing muscle tryptophan and preventing KYN accumulation through IDO-1 inactivation may lead to positive outcomes in various cases of muscle atrophy, including GC-related muscle atrophy.

## 6. Discussion and Perspectives

Skeletal muscle atrophy is caused by aging, stress, malnutrition, and the onset of chronic inflammation and metabolic disorders. In addition to muscle fiber damage, an imbalance between protein anabolism and catabolism is a major issue. It not only affects physical movement but also alters glucose metabolism and lipid deposition. Supplementation with protein, minerals, and some natural extracts may improve muscle function and protect against muscle wasting, but the effect may be insufficient in many cases [243,244,245]. Exercise provides widespread benefits across biological systems and helps maintain muscle mass [7]. However, it may be inapplicable for some patients and elderly persons. Thus, agents that prevent muscle atrophy and/or effectively stimulate muscle growth can address a significant unmet need.

GCs are critical in helping the body adapt to the rhythm of the human routine, from wakefulness to sleep, as well as, during pathological conditions, such as stress, infection, inflammation, and various diseases. Insulin and GC exhibit antagonistic roles in regulating glucose uptake and metabolism throughout the day [246]. The early morning peak in GC levels temporarily induces an increase in blood glucose and systemic glucose tolerance. The effect persists until insulin is released from pancreatic β-cells later in the morning through the afternoon. Insulin counteracts the effects of GCs by stimulating cellular glucose uptake and decreasing blood glucose levels. Circadian misalignment disrupts GC secretion patterns and impairs glucose tolerance, thereby increasing the risk of metabolic syndrome.

The interaction between insulin and GC plays a crucial role in the maintenance of muscle homeostasis. Insulin supports muscle generation and function by activating the PI3K/AKT/mTOR signaling pathway, which promotes glucose uptake and protein synthesis [247]. In contrast, GC interacts with the GR to initiate the transcription of atrogenes (e.g., FoxO3, MuRF1, Atrogin1), triggering protein degradation. GCs also suppress the expression and activation of IRS-1, a key component in the early steps of PI3K signaling. Furthermore, GCs enhance the levels of REDD1 and KLF-15, which directly inhibit mTORC1 and downstream signaling pathways involved in protein anabolism.

Under normal physiological conditions, myostatin enhances PTEN activity to regulate myoblast proliferation, thereby preventing muscle hypertrophy. However, overproduction of endogenous GC or excessive intake of synthetic GC disrupts the metabolic balance and negatively impacts muscle quality and quantity, particularly affecting glycolytic fast-twitch muscle fibers.

Drug discovery for skeletal muscle atrophy remains a significant challenge and requires reliable models that accurately represent physiological responses, particularly in the context of GC-associated myopathy. Although several compounds and biological molecules have successfully progressed to clinical trials, many have failed to meet expectations. The outcomes of these interventions are influenced by multiple factors including the potency of the inhibitors and the physiological characteristics of their targets.

Myostatin inhibitors are the most advanced therapies for the treatment of sarcopenia. Although myostatin inhibitors have shown promising effects in mouse models, their clinical efficacy in humans remains unclear. These inhibitors may increase muscle mass in some patients, but fail to restore functional movement. Additionally, some myostatin blockers lack specificity and inadvertently target BMP9/10, which is essential for muscle formation and function. Furthermore, myostatin levels are significantly lower in humans than in mice, which may limit the therapeutic benefits of myostatin inhibition. Another concern is that high GC accumulation in muscle tissues can counteract the effects of myostatin inhibition, further complicating the treatment outcomes.

Although the FoxO signaling pathway and atrogenic proteins are involved in the mechanism underlying GC-induced muscle atrophy, specific inhibitors targeting these pathways remain scarce and face significant limitations in terms of selectivity and efficacy for drug discovery. Moreover, blocking these catabolic pathways alone may be insufficient to restore muscle mass and function, as GCs not only promote proteolysis but also disrupt insulin signaling and glucose uptake.

SIRT6 inhibitors have shown the potential to increase the levels of GLUT4 and IGF-2, which improve glucose tolerance, stimulate the PI3K/AKT pathway, and simultaneously suppress the transcription of atrogenes. SIRT6 controls homeostasis by regulating the transcription of various genes, and cellular responses to SIRT6 inhibitors may vary in different tissues and situations. For example, in cases of malignancy, SIRT6 serves as both a tumor suppressor and an oncoprotein [178]. In muscle tissues, SIRT6 expression also plays a protective role against inflammation and cancer [248]. Thus, SIRT6 plays a central role in skeletal muscle biology. The effects of SIRT6 inhibition on inflammation- and GC-associated muscle atrophy should be further clarified using multiple models and analyses, especially in terms of genomic stability, because SIRT6 inhibition may broadly affect multiple genes related to inflammatory responses and protein metabolism. Additionally, the specificity of SIRT6 inhibitors is important. Other sirtuin isoforms, such as SIRT1, play contrasting roles to SIRT6 because SIRT1 prevents muscle wasting in response to GC [249] and cancer [250].

Activated GRs, in cooperation with LSD1 and NRF-1, drive the transcription of MuRF1, Atrogin1, and mTOR suppressor REDD1. LSD1 inhibitors block these effects and preserve the integrity of muscle proteins. Similar to SIRT6, LSD1 also plays a crucial role in epigenetic regulation. LSD1 controls fiber-type plasticity and metabolic switching in muscle tissues by exclusively suppressing oxidative phosphorylation in response to GC treatment [251]. Therefore, LSD1 inhibition may provide greater benefits in oxidative muscle fibers than in glycolytic muscle fibers. LSD1 inhibitors may specifically block the LSD1/GR protein–protein interaction and its downstream signaling, which induces proteolysis without interrupting the demethylase activity of LSD1.

The KYN pathway plays a complex role in muscle biology. Certain KYN metabolites, such as QUIN, exhibit neurodegenerative and proteolytic effects, whereas others, such as KYNA and NAM, support protein synthesis and provide energy to the muscle fibers. The inhibition of IDO-1, a key enzyme in the KYN pathway, prevents GC-induced muscle atrophy by increasing tryptophan availability for muscle growth and reducing ROS production, which drives oxidative stress and mitochondrial damage.

Skeletal muscle maintenance involves complex signaling pathways involved in protein production and glucose metabolism. Glycolytic fast-twitch muscle fibers are particularly sensitive to the atrophic effects of GC. Notably, GCs also protect muscle tissues from inflammation by suppressing NF-κB activation in the immune response, thereby facilitating wound healing and tissue regeneration. Unlike GR transactivation, which triggers protein degradation, GR transrepression supports muscle maintenance. Ideally, novel targets that prevent the adverse effects of GCs should selectively block GC-induced atrogene expression without interfering with its anti-inflammatory effects. Simultaneously, such inhibitors should induce protein synthesis and glucose uptake.

The specificity and affinity of the inhibitors are critical aspects of drug design. Inhibitors targeting epigenetic regulators, such as SIRT6 and LSD1, may face greater challenges owing to their potentially undesirable effects on the expression of various genes. Enzyme inhibition, such as IDO-1 inhibition, may offer a more predictable outcome. However, understanding the roles of SIRT6, LSD1, and IDO-1 in muscle biology requires further investigations. Despite the potency of their inhibitors in GC-induced muscle atrophy models, continuing exploration of the emerging targets may provide further opportunities for drug discovery to improve the quality of life of patients with myopathy and muscle loss caused by dysregulated endogenous GC production or prolonged use of synthetic GCs.

## Figures and Tables

**Table 1:** Recent blockers that may attenuate GC-induced muscle atrophy.

Targets	Compound	Structure	Activity	Refs.
11β-HSD1	- AZD4017 (ongoing phase II in IIH, NCT02017444)	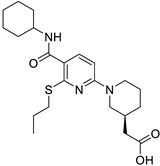	- Block cortisone conversion to cortisol - Prevent adverse effects of GC in muscle tissues - Increase lean muscle mass - (↓) FoxO1, MuRF1	[47,48]
Myostatin	- Taldefgrobep alfa (on-going phase III in SMA, NCT05337553 and phase II/III in DMD, NCT03039686)	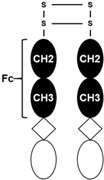 A fusion protein; Adnectin (binds to myostatin) and human IgG1-Fc	- Block myostatin binding to activin type I receptor - Activin IIb receptor antagonist - (↓) p-SMAD2/3	[61,62]
	- Apitegromab or SRK-015 (on-going phase III in SMA, NCT05156320, NCT05626855, and NCT05337553)	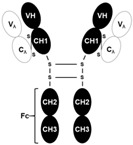 A full human IgG4	- Bind to pro-forms of myostatin - Prevent GC-induced muscle atrophy - (↓) p-SMAD2/3	[63,64]
Activin receptor	- Bimagrumab or BYM338 (on-going phase II in obesity, NCT05616013 and NCT05933499)	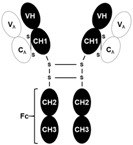 A full human IgG1	- Bind to activin receptor type IIA and IIB - Decrease body fat - Increase thigh muscle volume and fat-free body mass - - Prevent GC-induced atrogenes expression	[65,66]
FoxO1	- AS1842856	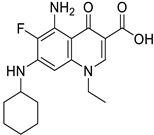	- FoxO1 inhibition - Cross-react with FoxO3 - (↑) Insulin sensitivity - (↓) Blood glucose and glucose tolerance - (↓) MuRF1, (↔) Atrogin1 - (↑) p70S6K	[67,68]
	- FOXO1-IN-3 (Compound **10**)	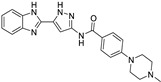		
FoxO3	- Carbenoxolone (available in some countries for the treatment of ulcers and GERD)	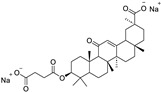	- 11β-HSD1 inhibitor - Cross-react with FoxO3 - Protect skeletal muscle from acute ischemia–reperfusion injuries - Attenuate FoxO3-induced chemotherapy resistance neuroblastoma	[69]
Vitamin D receptor	- Calcitriol and its derivatives (available for the treatment of SHPT, osteoporosis, and psoriasis)	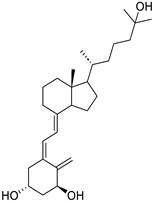	- (↓) MuRF1, Atrogin1 - Prevent GC-induced muscle atrophy	[70]

↑, increase; ↓, decrease; ↔, not change. Abbreviations: 11β-HSD1, 11β-hydroxysteroid dehydrogenase 1; IIH, idiopathic intracranial hypertension; GC, glucocorticoid; FoxO, forkhead box O; MuRF1, muscle RING finger 1; SMA, spinal muscular atrophy; IgG, immunoglobulin G; DMD, Duchenne muscular dystrophy; GERD, gastroesophageal reflux disease; SHPT, secondary hyperparathyroidism.

**Table 2:** Emerging targets for drug discovery for GC-induced muscle atrophy prevention.

Targets	Compound	Structure	Activity	Refs.
SIRT6	- SIRT6-IN-1	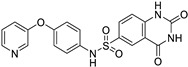	(↑) IGF2 (↑) GLUT-1,4 (↑) Glycolytic metabolism (↑) p-AKT (↓) FoxOs, Atrogin1, MuRF1	[17,173]
LSD1	- Pulrodemstat (CC-90011) (ongoing phase II in lung cancers, NCT03850067)	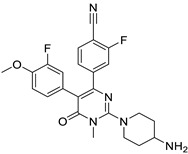	(↓) Myostatin, REDD1 (↓) FoxO3, Atrogin1 (↓) Polyubiquitin-C (↓) Autophagy (↓) NF-κB activity (↑) Myotube area (↑) Muscle proteins	[18]
	- GSK-LSD1 dihydrochloride	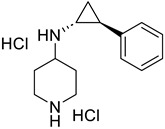		[174]
IDO-1	- Indoximod (D isomer of 1-MT) (ongoing phase II in brain tumors, NCT04049669)	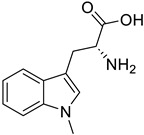	(↑) Serum tryptophan (↓) KYN (↑) KYNA (↓) ROS, TNF-α (↓) FoxO3, Atrogin1 (↑) p-AKT (↑) MHC	[19,175]
	- Navoximod (NLG-919) (completed phase I in solid tumors, NCT02048709 and NCT02471846)	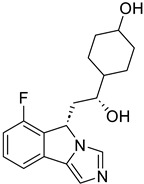		[176,177]

↑, increase; ↓, decrease. Abbreviations: SIRT6, Sirtuin 6 isoform; IGF, insulin-like growth factor; GLUT, glucose transporter; FoxO, forkhead box O; MuRF1, muscle RING finger 1; REDD1, regulated in development and DNA damage 1; NF-κB, transcription factors like nuclear factor-κB; IDO-1, indoleamine 2,3-dioxygenase 1; KYN, kynurenine; KYNA, kynurenic acid; ROS, reactive oxygen species; TNF-α, tumor necrosis factor-α; MHC, myosin heavy chain.
